# First-Stage Prostate Cancer Identification on Histopathological Images: Hand-Driven versus Automatic Learning

**DOI:** 10.3390/e21040356

**Published:** 2019-04-02

**Authors:** Gabriel García, Adrián Colomer, Valery Naranjo

**Affiliations:** Instituto de Investigación e Innovación en Bioingeniería (I3B), Universitat Politècnica de València (UPV), Camino de Vera s/n, 46008 Valencia, Spain

**Keywords:** gland classification, hand-crafted feature extraction, feature selection, hand-driven learning, deep learning, prostate cancer, histological image

## Abstract

Analysis of histopathological image supposes the most reliable procedure to identify prostate cancer. Most studies try to develop computer aid-systems to face the Gleason grading problem. On the contrary, we delve into the discrimination between healthy and cancerous tissues in its earliest stage, only focusing on the information contained in the automatically segmented gland candidates. We propose a hand-driven learning approach, in which we perform an exhaustive hand-crafted feature extraction stage combining in a novel way descriptors of morphology, texture, fractals and contextual information of the candidates under study. Then, we carry out an in-depth statistical analysis to select the most relevant features that constitute the inputs to the optimised machine-learning classifiers. Additionally, we apply for the first time on prostate segmented glands, deep-learning algorithms modifying the popular VGG19 neural network. We fine-tuned the last convolutional block of the architecture to provide the model specific knowledge about the gland images. The hand-driven learning approach, using a nonlinear Support Vector Machine, reports a slight outperforming over the rest of experiments with a final multi-class accuracy of 0.876±0.026 in the discrimination between false glands (artefacts), benign glands and Gleason grade 3 glands.

## 1. Introduction

Nowadays, prostate cancer is one of the most diagnosed types of cancer in the world, according to the last statistics published by the American Cancer Society (ACS) [1]. This study reveals that prostate cancer supposes the second cause of death related to cancer in men and the first type of cancer regarding the estimated new cases in 2019. In addition, ACS also situates this disease as one of the most common types of cancer in the USA concerning the general population, although it only affects men. On the other hand, the Spanish Society of Medical Oncology (SEOM) [2] exposes prostate cancer as a chronic disease due to their very high incidence and 5-year prevalence ratios. For this reason, it becomes necessary to carry out a fast and accurate diagnosis facilitating an early treatment to improve the quality of life of the patients with this chronic disease.

**Figure 1:**
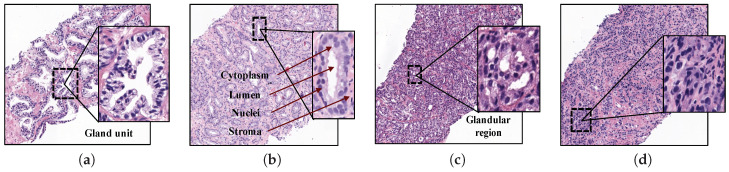
Samples of histopathological prostate tissues with different patterns according to the Gleason scale. (**a**) Grade 2 (normal); (**b**) Grade 3; (**c**) Grade 4; (**d**) Grade 5.

At present, the diagnostic procedure to detect prostate cancer is a very time-consuming task that is manually accomplished by pathologists or urologists. First, they carry out a rectal examination to find anomalies in the size of the prostate gland. The next step is to perform some analysis based on detecting specific antigens in the blood, such as Prostate-Specific Antigen (PSA) and Prostate-Cancer Antigen (PCA3). If all non-invasive tests are positive, the experts extract a sample of tissue and submit it to a preparation process composed of four phases: fixation, inclusion, cutting and staining. Once the samples are stained using the Hematoxylin and Eosin (H&E) pigment, the experts perform an in-depth examination under the microscope, or by computer if samples are previously digitised, in order to determine the definitive diagnosis. Thus, they make use of the Gleason classification system [3], which lies in assigning specific scores (from 1 to 5) to each tissue depending on the cancer severity (see Figure 1).

Particularly, Gleason grades 1 and 2 closely resemble the structure of normal tissues, since both have large and well-defined gland units. In addition, the lumens contain large areas surrounded by cytoplasmic complexes and usually by an epithelial multi-layer of nuclei. More specifically, Gleason pattern 1 corresponds to a well-differentiated (low grade) carcinoma, whereas Gleason pattern 2 corresponds to a moderately differentiated carcinoma. On the other hand, Gleason grades 3, 4 and 5 are related to cancerous tissues (from less to more severe). In particular, pattern 3 of Gleason is characterised by presenting dimensions of lumens and glands smaller and more circular. In addition, the cell density of the epithelial nuclei layer is lower in the pathological tissue. Gleason pattern 3 also corresponds to a moderately differentiated carcinoma. Regarding the Gleason grade 4, the tissues usually have glandular regions composed of the fusion of not well defined glands, but not gland units, whereas Gleason grade 5 is easily differentiated by the presence of a large number of scattered nuclei along the stroma. Gleason grades 4 and 5 correspond to a poorly differentiated carcinoma and an anaplastic carcinoma, respectively (see Figure 1).

### 1.1. Related Work

Currently, the biopsy analysis entails a considerable subjectivity level between pathologists, besides a large workload. For this reason, there are a lot of studies in the state of the art whose goal is to provide automatic models capable of reporting an initial indicative diagnosis from histopathological images. In most of these studies, the authors tried to perform computer-aided prognosis to discern between all Gleason grades [4,5,6,7] or simply between cancer and non-cancer [8,9,10]. Nevertheless, we propose for the first time a gland classification system exclusively focused on patterns corresponding to moderately differentiated carcinomas, i.e., patterns associated with Gleason grades 2 and 3, with the aim of helping the pathologists to reduce the workload and the subjectivity level when they try to diagnose this type of heterogeneous structures.

In the literature, we can mainly find three different image-processing strategies for encoding the relevant information from the histological images, which are widely implemented before addressing the classification stage: (i) construction of patches, (ii) detection of regions of interest (ROIs) and (iii) segmentation of gland units. Note that the kind of descriptors to extract the key information from the image is highly dependent on the selected approach.

Image processing associated with the construction of patches lies in dividing the whole-slide image (WSI) into different sub-images from which to extract discriminant information. It is the simplest approach since it evaluates the histological image without any previous identification of areas of interest. In [11], the authors implemented an initial sliding window protocol of 512×512 pixels with a 10% of overlap, from 12 histological scenes of 2295×4407 pixels. Later, using sub-regions of 100×100 pixels and applying textural and morphological descriptors, they achieved an accuracy of 0.79 in the distinction of stroma, normal and cancer tissues. In another study [12], the researchers analysed the fractal dimension of sub-bands derived from 1000 patches obtained with a magnifying factor of 400. Support Vector Machine (SVM) classifiers were used to finally achieve an accuracy of 0.86 in the Gleason classification system. On the other hand, in [7], the authors reported an overall accuracy of 98.3% in the Gleason grading of 268 images of 2448×3264 pixels. They applied a k-nearest neighbour (K-NN) classifier using textural features based on Local Binary Patterns (LBPs) and Gabor Filters. In a more recent study [10], the authors used 45 whole-slide images from which they composed sub-regions of three different dimensions: 512×512, 1024×1024 and 2048×2048 pixels. The best accuracy (0.98) was achieved by applying the surfacic granulometry descriptor on the 1024×1024 images and implementing an SVM classifier with a linear kernel.

The strategy based on ROI identification allows for obtaining more specific features because it is focused on relevant information. Notwithstanding, it entails a clear limitation since it requires either a manual identification, which supposes a tedious workload; or a previous automatic identification, which assumes an additional error during the process. This approximation is widely used in the state of the art because it allows for training a model exclusively based on relevant information. In [13], the authors tried to distinguish between stroma, benign epithelium, Gleason grade 3 and Gleason grade 4. They made use of 54 labelled tissue patches at 40× optical magnification from which they extracted morphological and textural features to provide an accuracy of 0.85 in the distinction of Gleason grade 3 and benign epithelium. Another study [5] made use of 268 colour images from representative areas of hematoxylin and eosin to address the Gleason grading. The researchers used colour, texture and morphometric features and achieved an accuracy of 0.81 implementing *k*-nearest neighbour and SVM classifiers. A recent study [14] developed a Gleason grading system directly using a data set of Gleason annotations that contained five tissue microarrays (TMAs) at 40× magnification. The authors divided each annotated TMA spot (3100×3100 pixels) in several patches (750×750 pixels), discarding those that had multiple annotations, with the aim of training a convolutional neural network (CNN) based on the MobileNet architecture. Then, the patches were reconstructed to the original spot size to evaluate them in terms of average recall. The reported results showed an accuracy of 0.7 concerning the Gleason grading task.

Regarding the strategy related to gland unit segmentation, it is similar to the previous since it also lies in identifying specific parts of the tissue that contain information of interest. Whereas in ROI-based detection, several patterns associated with different classes must be found, e.g., a group of poorly defined and fused glands (characteristic of the Gleason grade 4) or an accumulation of nuclei (typical of the Gleason grade 5), in the gland unit segmentation approach, a single type of structures is segmented (i.e., individual glands) along the whole tissue for then determining its class via machine-learning techniques. In [8], the authors applied a K-NN classifier on an imbalanced data set of 199 images and they reported an accuracy of 0.95 per image, only discerning between benign and malignant tissues. First, they used texture-based techniques to segment the prostate glands and they extracted several size and shape features from them to address the classification process. A more recent study [9] performed a tissue segmentation using 189 features based on the intensity and texture of the pixels. From the segmented regions of lumens and nuclei, the authors applied a total of 670 morphological features to detect prostate cancer and they reported an Area Under the ROC Curve (AUC) of 0.98 using a multi-view boosting classifier. In another recent study [15], the researchers made use of the Nuclei-Lumen Association (NLA) algorithm, proposed in [16], to carry out the gland unit segmentation. Then, the authors extracted features based on graphs and texture, as well as shape and orientation disorder of the glands, with the aim of distinguishing between cancerous and non-cancerous regions. They finally reported an AUC of 0.98 applying random forest classifiers on a database composed of 398 regions at 20× and 40× optical magnification. It is important to note that, in several studies of the state of the art such as [4,6,16], the authors implemented a similar strategy to ours since they used the lumen area as a starting point to address the gland unit segmentation. This approach also entails the segmentation of specific objects called “artefacts”, which are characterised by presenting a very similar structure and colour to the lumen elements. However, artefacts’ objects are differentiated because they are not surrounded by cytoplasm and epithelial nuclei components, unlike the lumens. In particular, Naik et al. [4] implemented a Bayesian classifier to detect the lumens and perform the gland segmentation from them. Nevertheless, the results showed important limitations because the gland boundaries were delimited by the cytoplasm structure, instead of by the nuclei components according to the stipulated in the medical literature [6]. From the previously segmented glands, the researchers applied morphological and textural features and reported an accuracy of 0.86 using a SVM classifier to distinct between benign and Gleason grade 3 tissues from a database of 44 images with an optical magnification of 40×. Otherwise, Nguyen et al. [16] developed the NLA algorithm to segment the gland units, which consisted of the linear union of the nuclei surrounding the lumen of a prostate gland. After the segmentation stage, the database was composed of 525 artefacts, 931 benign glands and 1375 cancer glands. The authors extracted a total of 22 features based on the context of each gland candidate and they reported an accuracy of 0.77 applying a SVM classifier to distinguish between the three classes. Note that, unlike the classification processes of the aforementioned works focused on a gland-unit level, the validation of the proposed methodologies was performed in a patch or image-wise way. The study performed in [16] is one of the few studies (along with ours) in which the predictive models were created and evaluated from the individual gland candidates, instead from regions. This strategy entails lower overall accuracy, but more reproducible and reliable results. The study performed in [17] is another example where the authors also carried out a gland classification discriminating between benign and pathological glands. However, in this case, the artefacts’ elements were not considered. The researchers extracted colour distributions, textural and structural features from the previously segmented 159 benign glands and 108 pathological glands; and they reported a final accuracy of 0.86 making use of SVM and boosted trees’ classifiers.

### 1.2. Contribution of This Work

We present in this paper an extended version of our recently published work [18], in which we made a comparison between traditional hand-driven learning and deep-learning techniques when performing a classification of benign and cancerous glands. The main difference with respect to all aforementioned studies lies in that we only focus on the identification and classification of individual glands what correspond to moderately differentiated carcinomas. This is because such individual glands, in spite of presenting a relatively homogeneous structure, also contain the necessary information to discern between normal and pathological tissues with a low grade of cancer, according to the stipulated by the specialists in pathological anatomy. Note that the discrimination between normal and cancerous tissues in a first stage could be decisive in the treatment of the patient.

In particular, we address the classification task from the individual gland candidates automatically segmented by means of the Locally Constrained Watershed Transform (LCWT) [19], which was applied for the first time on histological images in our previous work [20]. The evaluation of the LCWT algorithm performed in [20] reported better results, in terms of Jaccard index, than the popular NLA algorithm which, in turn, outperformed the methods developed in [4,21].

Regarding the approach related to the hand-driven learning, we incorporate in a novel way a hand-crafted feature extraction stage based on four global kinds of descriptors. We not only use morphological and textural descriptors as the majority of studies [4,8,11,13,15], but also descriptors related to the fractal dimension, like [5,12,22], and contextual features, similarly to [6,16]. Until now, each kind of descriptor had been implemented separately, but, in this paper, we build a hybrid feature vector able to encode all the relevant information included in the gland units.

In addition, taking into account some previous studies [14,23,24], which demonstrated the high viability of the deep-learning techniques in the field of the histopathological prostate image, we also include in this paper a network architecture to detect the first stage of the prostate cancer. It should be noted that, to the best of the author’s knowledge, this study is the first that purely uses the gland candidates as an input of the CNN, instead of using patches or regions with gland information.

Another contribution of this work is the presentation of a new database composed of prostate glands candidates, which is divided per categories into: artefacts, benign and pathological glands. Note that we provide two images per gland candidate: the bounding box enveloping the candidate and the same bounding box, but masked according to the outputs from the gland segmentation stage.

## 2. Materials

**Figure 2:**
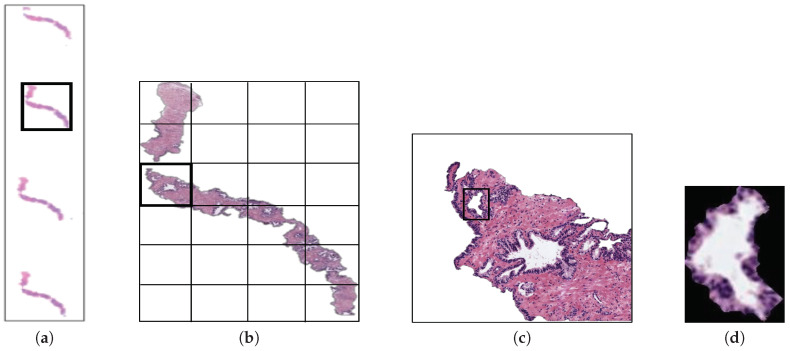
(**a**) example of a whole-slide image; (**b**) region of interest from which we perform the sliding window protocol; (**c**) sub-image of 1024×1024 pixels from which we address the segmentation task; (**d**) gland candidate achieved after applying the LCWT segmentation method.

The original database utilised in this paper consists of 35 whole-slide images (17 corresponding to healthy tissues and 18 containing tumour prostate areas) like the one shown in Figure 2a, which were pixel-wise annotated by an expert pathologist of the *Hospital Clínico Universitario de València*. The samples belong to 8 healthy patients and 17 with prostate cancer in an initial stage. From each slide, we initially address a simple pre-processing step in order to identify the bounding box that contains the region of tissue and remove those pixels that correspond to useless information (Figure 2a). Once the tissue of interest is identified, we implement a sliding window protocol to work with sub-images of reduced size that allow for improving the performance in terms of resolution and local information (Figure 2b). In particular, we divide the detected bounding box from the WSI in patches of 1024×1024 pixels, whose dimensions correspond to an optical magnification of 10×. Next, with the aim of improving the computational cost without affecting the study, we apply another simple step to discard those patches with less than 5% of tissue pixels in order to guarantee a minimum of useful information in each patch. In this way, the database from which we apply the gland segmentation methodology consists of 854 benign and 614 Gleason grade 3 sub-images of 1024×1024 pixels. It should be noted that this paper focuses on the building of predictive models from individual gland candidates, so the real database from which we work is composed of the gland candidates previously segmented with the aforementioned LCWT technique, as shown in Figure 2c. Therefore, after the segmentation stage, we obtain the final database for the classification task, which contains 3195 benign glands, 3000 cancerous glands of Gleason grade 3 and 3200 artefacts (false glands). Note that we randomly select some artefacts from the total obtained (22,045) with the aim of balancing the number of samples per class. The database, including original gland candidates and its automatic segmentation by the LCWT technique, can be downloaded from [25]. The resulting code of this work is publicly available in [26].

## 3. Methods

### 3.1. Flowchart

**Figure 3:**
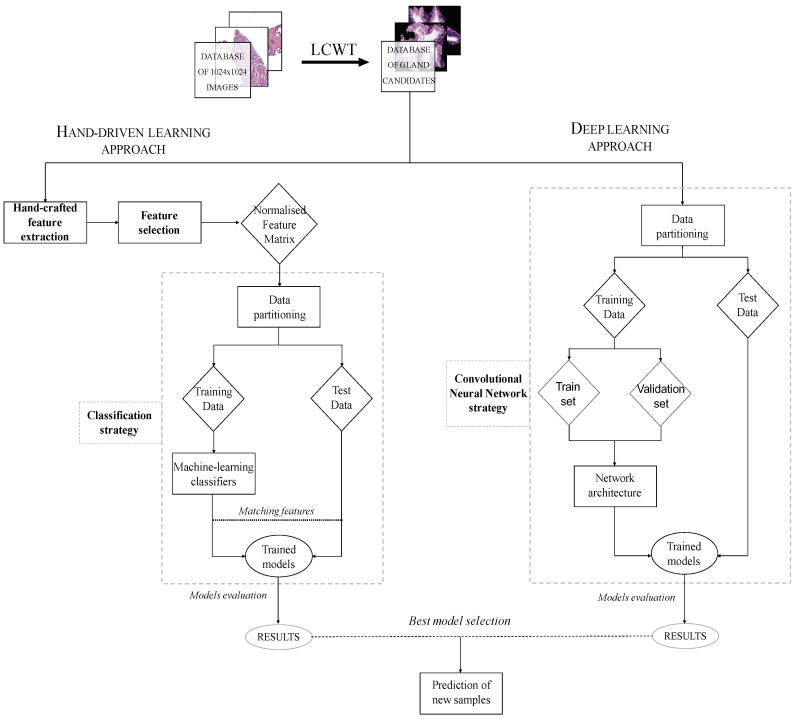
Flowchart in which we expose the two approaches performed from histopathological prostate images in order to address the gland candidates’ classification.

The methodology implemented in this paper is represented in the diagram exposed in Figure 3, in which we show the two strategies carried out from the segmented gland candidates. Regarding the hand-driven learning approach, we initially apply a hand-crafted feature extraction stage based on four families of descriptors. Then, we perform a statistical analysis to select the best features in terms of correlation and discriminatory ability. Once we have the normalised matrix of key features, we accomplish a data partitioning to divide the items into different sets in order to build robust and reliable predictive models during the classification stage. Finally, we report the results per gland achieved from two different optimised machine-learning classifiers. Concerning the deep-learning approach, we directly address the data partitioning from the previous segmented gland candidates images. In this way, we create 5 sets of images corresponding to such candidates, which constitute the input for the implemented convolutional neural network (CNN). Specifically, the CNN contains both the feature extraction phase, defined by the combination of four convolutional blocks, and the classification stage composed of two fully-connected layers. Finally, we compare the results achieved from the two approaches and use the best model to predict samples from new patients.

### 3.2. Background

**Figure 4:**
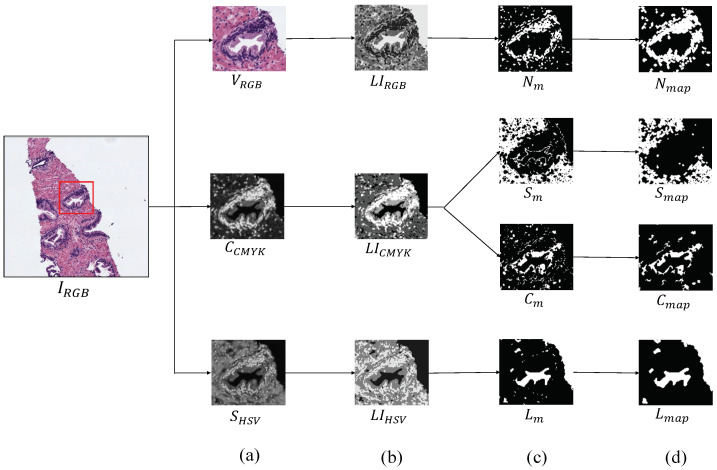
Process to obtain the binary map of each tissue component. (**a**) outputs after the colour space transformations; (**b**) labelled images achieved from the clustering stage; (**c**) masks obtained after the binarisation; (**d**) final maps of each tissue component.

The first step, before addressing the feature extraction stage, is to obtain separately the four main components that appear in the H&E prostate images, i.e., lumens, nuclei, cytoplasm and stroma, in order to compute different features from each component, as well as from the relation between them. Similarly to [15,16], we apply clustering algorithms based on the *k*-means technique to carry out the identification of each tissue component. However, unlike the previous studies that only used the RGB image as input for the clustering step, we use different colour spaces depending on the component mask that we want to extract. In particular, we make use of the saturation channel $S_{HSV}$ from the HSV (Hue, Saturation and Value) colour space to detect the lumen objects; the cyan channel $C_{CMYK}$ from the CMYK (Cyan, Magenta, Yellow and Key) colour space to identify the cytoplasm and stroma components and a reshaped RGB image $V_{RGB}$ to achieve the nuclei elements, as exposed in Figure 4a. Thus, from an initial RGB image $I_{RGB}$ of dimensions 1024×1024, we apply a reduction factor of 50% and perform the colour transformations to obtain each one of the four candidate maps. Once the different channels are computed, we select the number of clusters *k* in which the pixels of $I_{RGB}$ will be grouped. Specifically, we establish k=3 to obtain the map of lumen candidates $L_{m}$, as well as the maps of the cytoplasm $C_{m}$ and stroma $S_{m}$ candidates, whereas we set k=4 to acquire the nuclei map $N_{m}$. Thus, we apply the *k*-means algorithm on the $S_{HSV}$, $C_{CMYK}$ and $V_{RGB}$ images in order to group the pixels of each image into *k* different classes according to their intensity level. After the clustering stage, we obtain three labelled images LI (one from each colour space), whose pixels can take values from 1 to *k*, depending on the previous non-supervised classification of pixels (see Figure 4b). From here, we carry out a binarisation of each LI image by means of determining the value of the pixels associated with the tissue component of interest, as we show in Figure 4c. For instance, as it can be observed in Figure 4b, the darkest pixels of the $LI_{HSV}$ correspond to those relative to the lumen structures. Therefore, we can achieve the map of lumen candidates $L_{m}$ through applying Equation (1):

(1) $$L_{m}=\left\{\begin{array}{cc} 1, & \mathrm{if}\;LI_{HSV}=min\left(LI_{HSV}\right),\\ 0, & \mathrm{otherwise}. \end{array}\right.$$

It should be highlighted that we perform a post-processing stage from the outputs of the clustering, with the aim of removing the noise and obtaining the final binary maps: $L_{map},C_{map},S_{map},N_{map}$, exposed in Figure 4d. We apply different morphological operations according to the map of the tissue component that we want to obtain. In particular, in order to achieve the map of lumen candidates, we implement a filtering operation called *area opening* ($\gamma_{\lambda }^{a}$) Equation (2), from all set of pixels $X\subseteq L_{m}\subset R^{2}$. This morphological operation is defined by the union of all connected components of *X* whose area is greater than a number of pixels λ, according to the next equation:

(2) $$\gamma_{\lambda }^{a}\left(X\right)=⋃\left\{X_{i}\;|\;i\in I,\;Area\left(X_{i}\right)\geq \lambda \right\}.$$

After applying the area opening filter with a specific $\lambda_{lum}=20$ pixels, we also implement an operation of *dilation*, which is described as: $\delta_{B}\left(X\right)\;=\;X⊕B$, where *B* is a structuring element (SE) with radius r=1 and disk shape. Regarding the maps of cytoplasm $C_{m}$ and stroma $S_{m}$, we carry out a filtering phase based on an *opening* operation defined by $\gamma_{B}\left(X\right)=\left(X⊖B\right)⊕B$, followed by another *area opening*, in which we use $\lambda_{cyto}=\lambda_{str}=20$ pixels to discard the non-consistent sets of pixels. With respect to nuclei, we first apply a *dilation* using the SE *B* in order to emphasise the importance of the nuclei around the gland, and, later, we again implement an *area opening* $\gamma_{\lambda }^{a}$ also with $\lambda_{nuc}=20$ to discard those non-epithelial nuclei scattered across the stroma region.

Once the different binary maps of the tissue components are computed, we make use of them for two proposals. On the one hand, as inputs’ markers in order to address the segmentation stage by means of the LCWT algorithm, which was applied for the first time on histopathological images in our previous work [20]. In addition, on the other hand, to extract features from each tissue component, with the aim of distinguishing between artefacts, benign glands and pathological glands.

### 3.3. Hand-Driven Learning Approach

#### 3.3.1. Feature Extraction

This stage supposes one of the most remarkable novelties of this paper since, for the first time, we include information relative to the combination of 241 features from four different families of descriptors, which we detail below.

**Figure 5:**
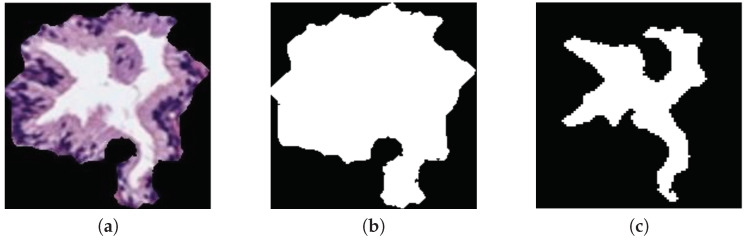
(**a**) segmented gland; (**b**) gland mask; (**c**) lumen mask.

**Morphological descriptors**. Along the state-of-the-art, several studies demonstrated the viability of this kind of features in the characterisation of the histological prostate image [4,8,9]. For it, we use a total of 20 features related to the morphology of the glands and their respective lumens, taking into account the differences about the Gleason grades detailed in Section 1. Specifically, we apply 10 features based on the shape and geometry of the gland candidates, and the same 10 features to extract the information associated with their lumens. Noticeably, the output of the LCWT algorithm correspond to an RGB image relative to the gland candidate, like the one shown in Figure 5a. Thus, in order to extract the morphological features, it is necessary to acquire the masks of the gland candidates, as well as the masks of their lumens. For it, let $Gland_{RGB}$ be a colour image of dimensions M×N, in which each pixel p(i,j) is denoted by the system Equation (3), where i=1,2,3…M and j=1,2,3,…N; we decompose the image into its three colour components (*R*, *G*, *B*) and sum them, according to Equation (4):

(3) $$p\left(i,j\right)=\left[\begin{array}{c} a\;b\;c \end{array}\right]\left[\begin{array}{c} R_{i,j}\\ G_{i,j}\\ B_{i,j} \end{array}\right],\;\mathrm{where}\;\left[a,b,c\right]\in \left[0,255\right],$$

(4) $$RGB_{s}=\sum_{i=1}^{M}\sum_{j=1}^{N}R_{i,j}+G_{i,j}+B_{i,j}.$$

Then, we apply the *Otsu* method [27] to identify an optimal threshold that allows for setting the pixels corresponding to the segmented gland to 1, and the rest to 0. Later, we carry out a simple filtering procedure based on an *area opening* with 4-connectivity and λ=20 pixels, followed by a flood-fill operation on background pixels. Once we obtain the gland mask $G_{mask}$ (represented by the white pixels in Figure 5b), we extract the lumen mask $L_{mask}$ (Figure 5c) by identifying the included lumen candidate inside the coordinates of the gland mask previously detected. Note that the name of each morphological feature is accompanied by *G* or *L* (gland or lumen) depending on the element under study. To explain the computed morphological features, we only define below those corresponding to the glands, but they are calculated in the same way for lumen masks:

- $G_{area}$. Number of pixels that contain a certain gland candidate.
- $G_{convexArea}$. Number of pixels in the region known as *convex hull* that are defined by the smallest convex polygon around the gland.
- $G_{eccent}$. Ratio of the distance between the centre of $E_{G}$ and its major axis length, where $E_{G}$ is the ellipse adjusted to the gland area with their same second moments.
- $G_{equivDiam}$. Diameter of a circle with the same area as the gland, defined by: $G_{equivDiam}=\frac{4*G_{area}}{\pi }$.
- $G_{extent}$. Ratio of pixels between $G_{area}$ and the area of the bounding box $G_{bBox}$ that contains the gland. It is computed as follows: $G_{extent}=\frac{G_{area}}{G_{bBox}}$.
- $G_{orientation}$. Angle between the *x*-axis and the major axis of $E_{G}$.
- $G_{perimeter}$. Number of pixels that describe the edge of the gland.
- $G_{solidity}$. Proportion of the pixels in the convex hull also included inside the area of the gland. It is described as: $G_{solidity}=\frac{G_{area}}{G_{convexArea}}$.
- $G_{roundness}$. Scalar that measures the compact character of the gland by: $G_{roundness}=\frac{R_{G}*G_{perimeter}}{G_{area}}$, where $R_{G}$ is the radius of the gland.
- $G_{compactness}$. Scalar indicating how round the gland is, according to: $G_{compactness}=\frac{G_{perimeter}}{\sqrt{G_{area}}}$.

**Figure 6:**
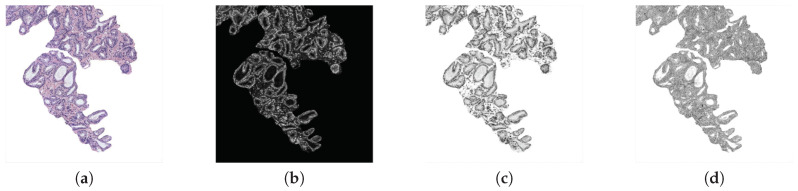
(**a**) RGB image; (**b**) cyan channel; (**c**) hematoxylin stain; (**d**) eosin contribution.

**Fractal analysis**. Several state-of-the-art studies, such as [5,9,12,22], used features based on different fractal dimensions to address the classification of histological prostate images. Particularly, we apply, for the first time on these kinds of images, a fractal analysis based on the Hurst exponent *H* [28]. Note that we make use of three different grey-scale images (cyan, hematoxylin and eosin) in order to take into account the contributions of different colour contributions (see Figure 6). Cyan is used because it is the channel in which the differences between the four tissue components can be better differentiated. In addition, we compute the hematoxylin and eosin colour images applying the colour deconvolution method proposed in [29], which was also implemented in other studies of the state of the art related with this research field, such as [10,30]. The colour deconvolution method allows for separating the contributions of each stain using the Optical Density (OD) parameter Equation (5), where *A* is the absorbance and $C_{s}$ is the concentration of a certain stain *s*:

(5) $$OD_{s}=A*C_{s}.$$

For each colour image, we compute the Hurst exponent *H*, which is related to the fractal Brownian motion (fBm). This fBm is a Gaussian, self-similar and non-stationary process $B_{H}\left(t\right)$ on [0,T], whose co-variance function Equation (6) was introduced in [31].

(6) $$\rho \left(s,t\right)=E\left[B_{H}\left(t\right)B_{H}\left(s\right)\right]=\frac{1}{2}{\left(|t\right|}^{2H}+{\left|s\right|}^{2H}{-|t-s|}^{2H}),\;\forall H\in \left[0,1\right],$$

where 0<s≤t and *H* corresponds to the aforementioned Hurst exponent, which governs the stochastic representation of the fBm and allows for measuring the complexity or tortuosity of the images. *H* is related to the fractal dimension according to H=E+1−FD, where *E* is the Euclidean dimension and FD the fractal dimension that takes higher values when the signal is more complex.

**Figure 7:**
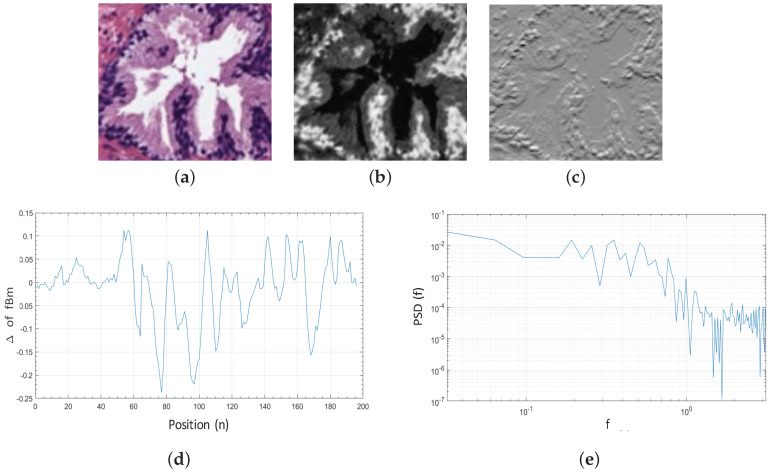
(**a**) original bounding box corresponding to an RGB gland candidate image; (**b**) cyan channel of the specific gland candidate; (**c**) increments of the fBm corresponding to the fractional Gaussian noise fGn; (**d**) 1D signal calculated from the fGn for m=1; (**e**) PSD of the increments of all rows from the gland candidate bounding box.

Additionally, being *X* the grey-scale bounding box of dimensions M×N that contains a specific gland candidate (Figure 7b), and taking into account that the fBm is a non-stationary process, it is more convenient to analyse the incremental process of the fBm (Figure 7c), i.e., the fractional Gaussian noise (fGn) defined as follows:

(7) $$G_{t}\left(a\right)=B_{H}\left(a+1\right)-B_{H}\left(a\right).$$

Once the fGn is computed, we obtain the discrete Fourier transform Equation (8), from the 1D signal d[n] Equation (9) calculated for each row m=1,2,3,…,M, where n=1,2,3,…,N−1. We expose in Figure 7d an example of a 1D signal for m=1:

(8) $$D\left[a\right]=\sum_{n=0}^{N-1}d\left[n\right]e^{\frac{-j2\pi an}{N}},$$

(9) d[n]=X[m,n+1]−X[m,n].

From the fGn function and being $D^{\prime }\left[a,b\right]$ a 2D matrix composed of the *M* rows corresponding to all 1D signals previously achieved, we calculate the average Power Spectral Density (PSD) as follows:

(10) $$PSD\left[a\right]=\frac{1}{M}\sum_{t=0}^{M-1}D^{\prime }\left[a,b\right].$$

In a log–log scale, the PSD function of the fGn corresponds to a line of slope 1−2H, which we obtain by means of linear regression, as we expose in Figure 7e. The Hurst exponent *H* is finally calculated to determine if the pixels of the gland candidates follow purely random patterns or keep underlying trends. In particular, we consider L = 5 directions to calculate the Hurst exponent along each one of them: $H=\{H^{0{}^{\circ}},H^{30{}^{\circ}},H^{45{}^{\circ}},H^{60{}^{\circ}},H^{90{}^{\circ}}\}$, with the aim of analysing the different patterns in the orientation of the glands. Note that *H* is extracted from the cyan, hematoxylin and eosin channels, so 15 features related to the fractal dimension ($H_{cyan}$, $H_{hmtx}$ and $H_{eosn}$) are finally computed.

**Texture descriptors**. In this work, we make use of two kinds of descriptors for encoding the textural information related to the artefacts, benign and pathological glands. On the one hand, we use Gray-Level Co-occurrence Matrix (GLCM), similarly to Leo et al. [15], who calculated a 18×18 co-occurrence matrix to obtain information about the glands’ orientation. In other studies, such as [11,13,32], the authors also applied GLCM-based techniques but on histological regions, instead of gland units. On the other hand, we use Local Binary Patterns (LBP) to extract local intensity changes of the gland candidates, unlike other works which used LBP to segment different tissue structures [9,30] or discriminate between cancerous and non-cancerous patches [7,10]. Note that, in this case, we also use the cyan, hematoxylin and eosin channels to compute a total of 186 textural features.

**Figure 8:**
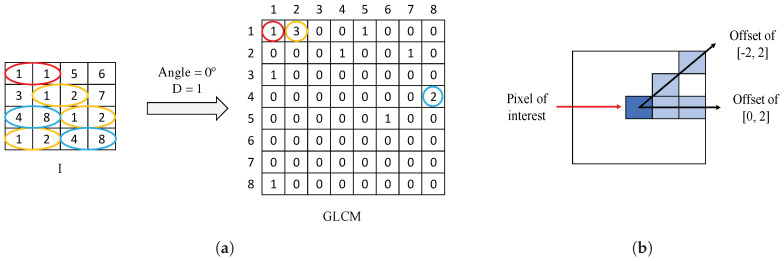
(**a**) example of GLCM achieved from a certain image *I* using an offset of [0,1]; (**b**) illustration of the two offset implemented in this paper to create each GLCM.

Gray-Level Co-occurrence Matrix (GLCM) is a matrix of frequencies, in which it is represented (on the (i,j)-position) the number of times that a pixel with an intensity value *i* is adjacent to another pixel whose intensity value is *j*. During the GLCM creation, we specify the number of adjacent pixels *D* that must have the intensity value j=i, as well as the direction (angle) in which such pixels are considered adjacent. In Figure 8a, we show an example of a GLCM obtained from a specific image *I* with an angle of 0° and D=1 pixels. Particularly, we apply a number of adjacent pixels D=2 and establish two different directions corresponding to angles of 0° and 45°, with the aim of considering the trend in the orientation of the gland candidates. The two directions are represented by an offset of [0,2], relative to the angle of 0°, and another offset of [−2,2], corresponding to an angle of 45°, as shown in Figure 8b. Note that the dimensions of the GLCMs computed in this paper for each colour image are always 8×8 pixels.

Once the GLCM is obtained, we normalise it by means of Equation (11), where $sGLCM=GLCM+GLCM^{T}$ corresponds to the symmetric GLCM [33]:

(11) $$nGLCM=\frac{sGLCM}{\sum sGLCM}.$$

From a certain normalised $nGLCM_{r}^{s}$, corresponding to an offset *s* and a colour image *r*, we extract 21 different features. The fact of using two offset values and three colour images allows for obtaining a total of 126 features related to the GLCM for each gland candidate. We detail below the 21 different variables, for an offset of [0,2], denoted by *0°*, and a colour image corresponding to the cyan channel *C*. Remember that these features are equally extracted from the rest of offsets and colour images.

- Homogeneity. It reaches high values when the occurrence is focused along the normalised GLCM diagonal. Being p(i,j) the probability of occurrence of the grey values *i* and *j*, the homogeneity is calculated according to:
  (12) $$Homo_{nGLCM_{C}^{0}{}^{\circ}}=\sum_{i,j}\frac{p(i,j)}{1+|i-j|}.$$
- Contrast. It measures the local variation of a certain image. It is the opposite to the homogeneity and it is computed by:
  (13) $$Cont_{nGLCM_{C}^{0}{}^{\circ}}=\sum_{i,j}{\left|i-j\right|}^{2}\;p\left(i,j\right).$$
- Energy. The energy, a.k.a. the angular second moment, takes small values when all inputs are similar. It is denoted as follows:
  (14) $$Ener_{nGLCM_{C}^{0}{}^{\circ}}=\sum_{i,j}p{\left(i,j\right)}^{2}.$$
- Correlation. The correlation indicates how much similar information provides a pixel over the whole image regarding its neighbour. It is denoted by:
  (15) $$Corr_{nGLCM_{C}^{0}{}^{\circ}}=\sum_{i,j}\frac{\left(i-\mu i)(j-\mu j)p(i,j\right)}{\sigma_{i}\sigma_{j}}.$$
- Entropy. It is a measure related to the uniformity of image. The entropy takes small values when the inputs of nGLCM are close to 0 or 1. It follows the next equation:
  (16) $$Entr_{nGLCM_{C}^{0}{}^{\circ}}=\sum_{i,j}-p(i,j)\ln(p(i,j)).$$
- Mean(μ). This feature corresponds to an average by columns of the grey values of the 8×8nGLCM. Thus, we obtain N=8 values from j=1,2,3,…,8 columns, according to:
  (17) $$\mu_{nGLCM_{C}^{0}{}^{\circ}}^{j}=\frac{1}{N}\sum_{i=1}^{N}p(i,j).$$
- Standarddeviation(σ). Similarly to the previous variable, here we also obtain eight values relative to the standard deviation of the 8×8nGLCM calculated by columns:
  (18) $$\sigma_{nGLCM_{C}^{0}{}^{\circ}}^{j}=\sqrt{\frac{1}{N-1}\sum_{i=1}^{N}{\left|p\left(i,j\right)-\mu \right|}^{2}}.$$

Local Binary Patterns (LBP) are also used in order to recognise local textures, as well as identify specific shapes in the images of gland candidates. The basic LBP operator proposed in [34] consists of computing the difference between the value of the pixel of interest and the value of its neighbours. The pixels under study are binarised to 0 or 1 depending on whether the resultant values are negative or positive, as follows:

(19) $$LBP_{P,R}\left(i,j\right)=\sum_{p=0}^{P-1}s\left(g_{p}-g_{c}\right)2^{p},\;s\left(x\right)=\left\{\begin{array}{cc} 1, & \mathrm{if}\;\;x\geq 0,\\ 0, & \mathrm{if}\;\;x\leq 0, \end{array}\right.$$

where *P* is the number of neighbour pixels with a grey value $g_{p}$ inside of a circle of radius *R*, respecting to the central pixel p(i,j), whose grey value is $g_{c}$. In this way, from the created binary string, it is possible to obtain the new value of the pixel of interest by performing a conversion to a decimal value. However, we do not implement the basic LBP, but we use the $LBP_{P,R}^{riu2}$ operator Equation (20), proposed by Ojala et al. in [35], which is characterised by being uniformly invariant to rotation transforms for grey-scale images. $LBP_{P,R}^{riu2}$ operator allows for obtaining P+2 different output values, taking into account the demonstrations exposed in [36]. Specifically, we use P=8 and R=1, so we extract 10-bin $LBP_{P,R}^{riu2}$ histograms from each colour image corresponding to a certain gland candidate. Since we make use of the cyan, hematoxylin and eosin contributions, we extract a total of 30-bin histograms uniformly invariant to rotation transforms:

(20) $$LBP_{P,R}^{riu2}\left(i,j\right)=\left\{\begin{array}{cc} \sum_{p=0}^{P-1}s(g_{p}-g_{c}), & \mathrm{if}\;\;U(LBP_{P,R})\leq 2,\\ P+1, & \mathrm{otherwise}, \end{array}\right.$$

where

(21) $$U\left(LBP_{P,R}\right)=\left|s\left(g_{p-1}-g_{c}\right)-s\left(g_{0}-g_{c}\right)\right|+\sum_{p=1}^{P-1}|s\left(g_{p}-g_{c}\right)-s\left(g_{P-1}-g_{c}\right)|.$$

In addition, we make use of the operator called Rotational Invariant Local Variance (VAR) Equation (22), which is commonly implemented in combination to the $LBP_{P,R}^{riu2}$ since it is invariant against contrast changes:

(22) $$VAR_{P,R}\left(i,j\right)=\frac{1}{P}\sum_{p=0}^{P-1}{\left(g_{p}-\mu \right)}^{2},\;\mathrm{where}\;\mu =\frac{1}{P}\sum_{p=0}^{P-1}g_{p}.$$

From the $LBP_{P=8,R=1}^{riu2}$ and $VAR_{P=8,R=1}$ images of dimensions M×N, we extract the LBP variance (LBPV) histogram Equation (23), which was proposed by Guo et al. [37] and consists of the accumulation of the $VAR_{P,R}$ value for each $LBP_{P,R}^{riu2}$ label, according to:

(23) $$LBPV_{P,R}\left(k\right)=\sum_{i=1}^{M}\sum_{j=1}^{N}w(LBP_{P,R}\left(i,j\right),k),\;k\in \left[0,K\right],$$

where

(24) $$w\left(LBP_{P,R}\left(i,j\right),k\right)=\left\{\begin{array}{cc} VAR_{P,R}\left(i,j\right), & \mathrm{if}\;LBP_{P,R}\left(i,j\right)=k,\\ 0, & \mathrm{otherwise}. \end{array}\right.$$

**Figure 9:**
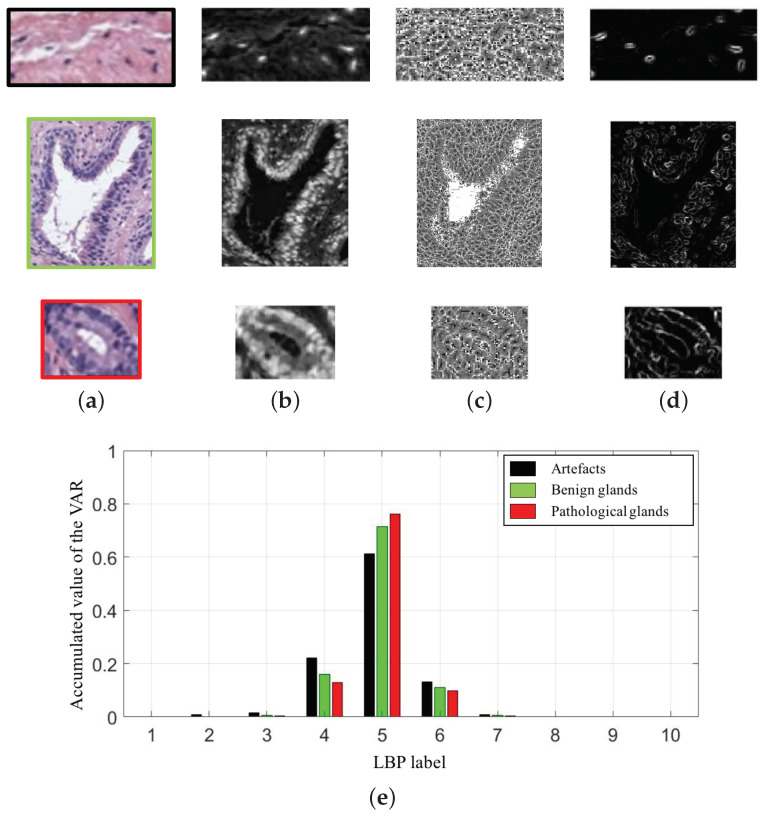
(**a**) gland candidates related to an artefact, a benign and a pathological gland highlighted in black, green and red, respectively; (**b**) cyan channel of the the gland candidates; (**c**) $LBP_{8,1}^{riu2}$ image; (**d**) $VAR_{8,1}$ image; (**e**) 10-bin histograms of the $LBPV_{8,1}$ after combining the images (c–d).

In particular, we compute 10-bin LBPV histograms for each colour image (cyan, hematoxylin and eosin). In Figure 9, we show an example of the $LBPV_{8,1}$ variable extracted from the cyan channel of the three types of gland candidates, i.e., an artefact, a benign gland and a Gleason grade 3 gland. Therefore, taking into account the 30-bin histograms corresponding to the $LBP_{8,1}^{riu2}$ and the 30-bin histograms relative to the $LBPV_{8,1}$, we accomplish a total of 60 features related to the LBP descriptor.

**Contextual features**. Nguyen et al. [6,16] used structural features to address a classification problem from two different approaches. Particularly, in [6], they extracted 15 structural variables from the previous detected glandular regions. However, it should be noted that the study was performed exclusively making use of 82 ROIs carefully selected by hand, whereas, in this paper, we address an end-to-end approach from all of the images of the database. In this way, a total of 20 hand-crafted features related to the context of each gland candidate image are computed and group below three different sets of variables, as we detail below.

The first set contains 10 features related to the nuclei components:

- $nuclei_{BB}^{num}$. It corresponds to the quantity of nuclei elements inside of the bounding box of the segmented gland $G_{bBox}$. Being $NE=\{p_{1},p_{2},\ldots ,p_{T}\}$ a certain individual nuclei element composed of *T* connected pixels $p_{i}$ with 8-connectivity, we compute:
  (25) $$nuclei_{BB}^{num}=\sum NE,\;\mathrm{where}\;\;NE\subseteq G_{bBox}.$$
- $nucleiRatio_{BB}^{num}$. We calculate the ratio relative to the number of nuclei elements and the area of the $G_{b}Box$ ($G_{bBox}^{area}$), according to:
  (26) $$nucleiRatio_{BB}^{num}=nuclei_{BB}^{num}/G_{bBox}^{area}.$$
- $nuclei_{Gland}^{num}$. It is similar to the first variable, but, here, we calculate the number of nuclei objects inside the gland candidate area $G_{area}$, instead of inside the $G_{bBox}$ area.
  (27) $$nuclei_{Gland}^{num}=\sum NE,\;\;\;\;\;\;\mathrm{where}\;\;NE\subseteq G_{area}.$$
- $nucleiRatio_{Gland}^{num}$. Similarly to the second feature, we also calculate the ratio of nuclei objects, but, with respect to the gland candidate area, as we expose below:
  (28) $$nucleiRatio_{Gland}^{num}=nuclei_{Gland}^{num}/G_{area}.$$
- $nucleiRatio_{Gland-BB}^{num}$. In this case, we compute the ratio between the number of nuclei elements inside the area gland in relation to the number of nuclei elements inside the bounding box, according to:
  (29) $$nucleiRatio_{Gland-BB}^{num}=nuclei_{Gland}^{num}/nuclei_{BB}^{num}.$$

It is remarkable that the rest of variables related to the nuclei components are calculated in the same way as before, but taking into account the number of pixels of the nuclei elements, instead of the number of nuclei elements themselves. Thus, being $pix=\sum_{i=1}^{T}p_{i}$, where $p_{i}\subseteq NE$, we acquire the next: $nuclei_{BB}^{pix}$, $nucleiRatio_{BB}^{pix}$, $nuclei_{Gland}^{pix}$, $nucleiRatio_{Gland}^{pix}$ and $nucleiRatio_{Gland-BB}^{pix}$.

Regarding the second set, it contains five variables relative to the cytoplasm structure. Specifically, we obtain these features in a similar way as the previous five, but, in this case, we compute the variables from the pixels corresponding to the cytoplasm component in order to obtain: $Cyto_{features}=\left\{cyto_{BB}^{pix},\;cytoRatio_{BB}^{pix},\;cyto_{Gland}^{pix},\;cytoRatio_{Gland}^{pix},\;cytoRatio_{Gland-BB}^{pix}\right\}$.

Finally, the third set corresponds to five contextual features associated with specific relations between the lumen, nuclei and cytoplasm components, as we detail below:

- $Ratio_{L-G}^{pix}$. This feature makes reference to the proportion between the lumen and gland areas, in terms of number of pixels. It is computed as follows:
  (30) $$Ratio_{L-G}^{pix}=L_{area}/G_{area}.$$
- $\mu_{L-Edge}$. We calculate the average distance between the centroid of a lumen and the pixels of its edge, using the Euclidean distance according to:
  (31) $$\mu_{L-Edge}=\frac{\sum_{i=1}^{N}\sqrt{{\left(c_{x}-x_{i}\right)}^{2}+{\left(c_{y}-y_{i}\right)}^{2}}}{N},$$
  where *N* is the total number of pixels of the lumen edge, $\left(c_{x},c_{y}\right)$ are the coordinates corresponding to the centroid of the lumen and $\left(x_{i},y_{i}\right)$ the coordinates of the pixel *i* relative to the lumen edge.
- $\sigma_{L-Edge}$. We also compute the standard deviation of the distance between the centroid and the edge of each lumen, by means of:
  (32) $$\sigma_{L-Edge}=\frac{1}{N-1}\sqrt{\sum_{i=1}^{N}|v_{i}-\mu_{L-Edge}{|}^{2}},$$
  where
  (33) $$v_{i}=\sqrt{{\left(c_{x}-x_{i}\right)}^{2}+{\left(c_{y}-y_{i}\right)}^{2}}.$$
- $Tor_{C-N}$. To compute this feature, first, we acquire a toroid region by subtracting the masks relative to the gland and lumen candidates, $G_{mask}$ and $L_{mask}$, respectively, according to $Toroid=(G_{mask}-L_{mask})$. Then, we calculate the number of pixels associated with the nuclei and cytoplasm masks, $N_{mask}$ and $L_{mask}$, inside the toroid region, as follows:
  (34) $$Tor_{C-N}=\sum Toroid\cap (C_{mask}+N_{mask}).$$
- $ToroidRatio_{C-N}$. It corresponds to the ratio between the number of pixels of the cytoplasm and nuclei masks inside the Toroid with respect to the area of such region $Tor_{area}$. It is defined by:
  (35) $$RatioTor_{C-N}=Tor_{C-N}/Tor_{area}.$$

#### 3.3.2. Feature Selection

**Figure 10:**
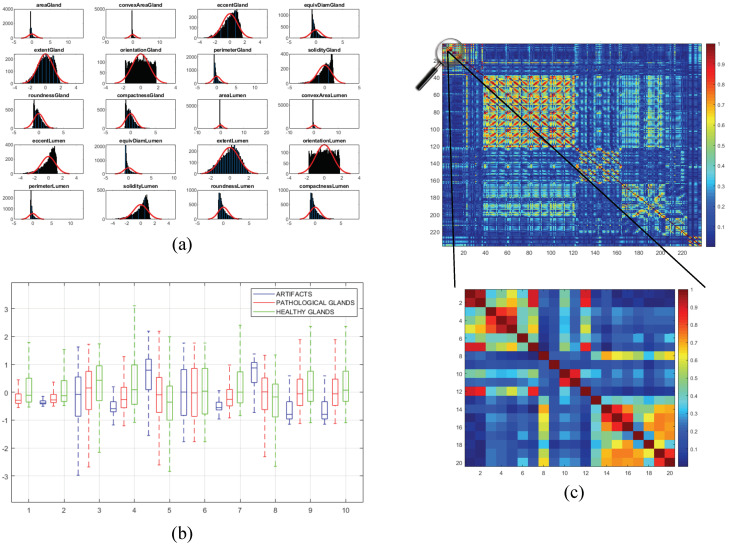
(**a**) bar chart corresponding to the distribution of the first 20 variables overlapped on the Gaussian bell represented in red; (**b**) box plot relative to the discriminatory ability of the first 10 variables; (**c**) correlation matrix that visually shows the independence level between pairs of variables.

Once the 241 variables are computed and stored, we address an exhaustive statistical analysis based on parametric and non-parametric tests to select the most relevant features, in terms of independence between pairs of variables and dependence between each feature and the ground-truth label. The first step is to normalise the variables, for assigning the same relevance to each of them, according to the *z-score* parameter described by: $z_{i}=\frac{x_{i}-\mu }{\sigma }$. In this way, $z_{i}$ is the normalised number of the $x_{i}$ value for a specific variable with mean μ and standard deviation σ. After normalising the variables, we perform the non-parametric *Kolmogorov–Smirnov test* to carry out a hypothesis contrast in which the null hypothesis $H_{0}$ maintains that the variables follow a normal distribution N(0,1). In Figure 10a, we represent the histogram concerning the first 20 computed variables over a Gaussian function, a.k.a. Gauss bell, which characterises the normal distribution N(0,1) to visually analyse the differences between both distributions.

Depending on the previous statistical test, we perform another contrast hypothesis with the aim of analysing the discriminatory ability of each variable with respect to the class. Thus, we carry out a comparison of means making use of the *ANOVA* test (ANalysis Of VAriance) if the variable follows a distribution N(0,1), or a comparison of medians using the *Kruskal–Wallis* test if the variable does not follow a normal distribution. In this way, set a significance level $\alpha =10^{-6}$ corresponding to a confidence value of 99.9999%, we compare α with the p-value obtained from the *ANOVA* or *Kruskal–Wallis* test to determine the independence level between each variable and its class. Finally, if p-value ≤α, we reject the null hypothesis $H_{0}$ that holds the independence variable-class because there is a significant evidence to ensure that the feature under study is dependent on the class. Thus, this variable is selected due to its high discriminatory ability. On the contrary, we discard the variables whose p-value is greater that α since they are not dependent on the class. In Figure 10b, we show the box plot corresponding to the first 10 variables in order to visually demonstrate the differences between the values obtained for each feature depending on its ground-truth labels.

In addition, we perform a third hypothesis contrast to analyse the independence level between pairs of variables. We discard the features that are correlated with others with the aim of avoiding redundant information. For it, we calculate the correlation coefficient *r* and the p-value from the correlation matrix to remove those variables that meet both p-value ≤α and |r|≥0.95. In Figure 10c, we show an example of the correlation matrix obtained during the feature selection stage. Finally, from the 241 variables, we obtain a total of 136 relevant features that we list below in Table 1.

#### 3.3.3. Classification Strategy

**Data partitioning**. From the computed data matrix related to the previously selected hand-crafted features, we divide the different items (rows) into 5 data sets taking into account diverse criteria. On the one hand, we include a similar number of items in each set (fold), attending to the class of such items to create balanced groups. On the other hand, note that, to the best of the author’s knowledge, we are the first who perform a partitioning separating the data according to the medical history of the patients. Thus, all the values collected from the different gland candidates corresponding to the same patient are stored in the same fold. Once the five folds are built, we carry out a nested cross-validation strategy to remove the randomness effect in the data partitioning. First, we perform an external *5-fold cross-validation* to train the models using four partitions and validate their performance by means of the remaining fold. This process is repeated five times to ensure that all the samples are used to train and test. Additionally, in each iteration, we also apply an internal *10-fold cross-validation* to optimise the parameters of the classifiers using the 10% of the training data as a validation set.

**Figure 11:**
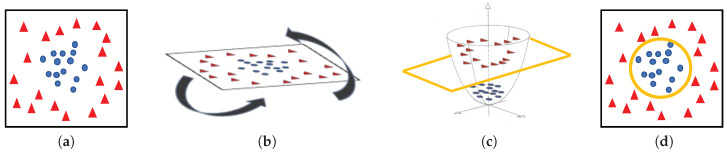
(**a**) original input space; (**b**) rotation of the plane of data; (**c**) 3D space transformation where data are linearly separable; (**d**) representation of the classification boundary on the 2D plane.

**Machine-learning classifiers**. In order to address the classification stage through hand-driven learning methods, we make use of two different classifiers widely utilised in the literature [6,9,10,15]. Specifically, we optimise a Support Vector Machine (SVM) classifier, which allows for finding the optimal hyperplane *h* that separates two regions of the input space maximising the distance between two *support vectors*, one of each class [38]. In particular, due to the complexity of the problem under study, we make use of a quadratic kernel to address the multi-class classification from a nonlinear approach. Note that kernels allow for projecting a *D*-dimensional input space to another M>D, according to $\phi =R^{D}\to R^{M}$, with the aim of performing a space transformation in which the data can be linearly separated, as shown in Figure 11.

**Figure 12:**
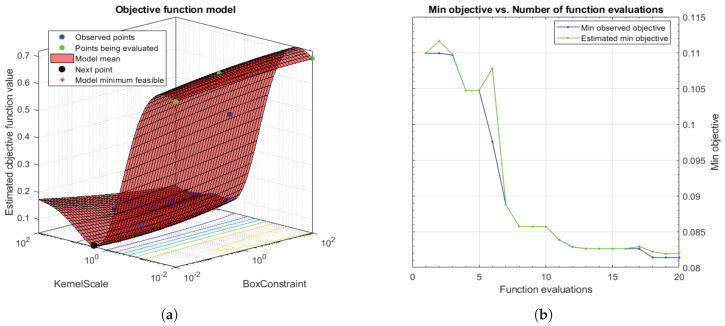
(**a**) 3D objective function that shows how the model find the optimal minimum modifying the hyperparameters *C* and γ; (**b**) diagram that shows how to reach the minimum objective as the number of time increases.

Taking into account that SVMs are non-parametric classifiers that were originally formulated to face binary problems, we divide the initial multi-class problem (artefacts, healthy glands and pathological glands) into two binary sub-problems (artefacts vs. glands and healthy glands vs. pathological glands), through applying a *Onevs.One* (OvO) strategy. In addition, the polynomial order and the binary type of classification, SVM classifiers also allow for optimising other hyperparameters such as the *box constraint* *C* and the *kernel scale* γ. The first helps to prevent overfitting by means of managing the maximum penalty imposed on margin-violating observations. Thus, the higher value of *C* the fewer support vectors are assigned and the overfitting is smaller, but it also entails longer training times. Regarding the γ hyperparameter, it allows for defining the influence of a single training example. High values of γ allow for decreasing the computational time, but it also implies that the weights of the observations closest to the decision boundary have greater importance than the rest. For these reasons, we specify the same range of values for both parameters $C\in [10^{-2},10^{2}]$ and $\gamma \in [10^{-2},10^{2}]$, in order to find a trade-off solution between computational cost and robustness of the models. In particular, we make use of the Bayesian optimisation algorithm that attempts for minimising a scalar objective function through evaluating the expected amount of improvements and modifying the behaviour when the classifier estimates that a local area is overexploited. Thus, making use of the aforementioned internal cross-validation, we find the optimal *C* and γ values, as shown in Figure 12, and use them to train a new classification model composed of all 4-fold training data (see Section 3.3.3). In this way, it is possible to consider the validation data for the models construction. For the specific case of Figure 12, the best performance of the model is reached at epoch 18 for C=99.30 and γ=28.88.

**Figure 13:**
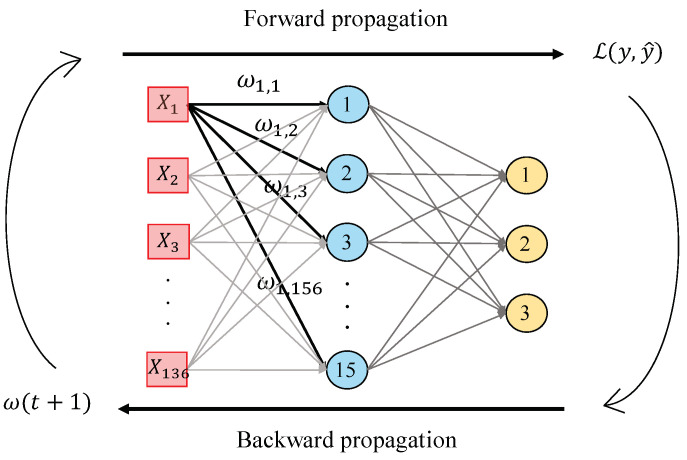
Illustrative example showing the forward-backward propagation algorithm from an MLP architecture composed of one hidden layer with fifteen neurons and an output layer with three classes.

In this work, we perform a second hand-driven learning classifier using a feed-forward neural network based on the Multi-Layer Perceptron (MLP). According to [39], MLP is one of the best models in terms of pattern recognition from hand-crafted features. This type of neural network consists of a set of logistic regressions in which the output of each is the input of the next one. In this way, MLP algorithm is based on a forward-backward propagation model that allows for updating the weights and bias depending on the loss function error obtained during the forward propagation step for each epoch. In this work, we design an MLP architecture with one hidden layer and fifteen neurons, as shown in Figure 13, taking into account that more hidden layers would provide considerable overfitting. The inputs $X=\{X_{1},X_{2},X_{3},\ldots X_{136}\}$, correspond to the values of the variables from each sample, i.e., the values of each item from the normalised data matrix. Particularly, we perform the update of the weights ω making use of the gradient descent algorithm, but also considering a momentum α=0.9 and an adaptive learning rate backpropagation, according to Equation (36):

(36) $$\omega \left(t+1\right)=\alpha \omega \left(t\right)+\eta \alpha \frac{d}{d\omega (t)}L(y,\hat{y}),$$

where ω(t) initially takes low random values and $L(y,\hat{y})$ is the loss function corresponding to the categorical cross-entropy that measures the performance of the classifier according to Equation (37):

(37) $$L\left(y,\hat{y}\right)=-\sum_{i}y_{i}\;log\left({\hat{y}}_{i}\right),$$

where $y_{i}$ corresponds to the output (i.e., the prediction) achieved from the model after each epoch, and ${\hat{y}}_{i}$ is the ground-truth label corresponding to the target of the training data. In addition, $\eta =10^{-3}$ is the initial learning rate that varies for each epoch following the next low: if the loss function error decreases up to the goal, the learning rate is increased by a factor $\mu_{u}=1.5$, whereas, if the performance increases by more than m=1.04, then the learning rate is decreased by a factor $\mu_{d}=0.5$.

Note that, in this case, unlike the strategy followed by the SVM approach, we do not perform internal cross-validation techniques since we initially define all of the hyperparameters. However, we also include a validation subset for each training set in order to analyse the behaviour of the model and supervising if overfitting phenomenon appears along the epochs. In addition, we set the maximum number of epochs N=1000 and impose a stop criterion to end the training process if the performance of the neural network does not improve after 20 epochs.

### 3.4. Deep-Learning Approach

#### Convolutional Neural Network Strategy

**Data partitioning**. In this case, we directly construct the predictive models from the previous segmented gland candidates images since the computed CNN allows for automatically extracting the key features, by means of the convolutional layers contained in the base model. Similarly to the hand-driven learning strategy, we perform the data partitioning based on the medical history of each patient and also applying an external 5-fold cross-validation to evaluate the performance of the models. However, instead of carrying out an internal cross-validation, we define a random subset of learning instances to validate the weights optimisation during each training iteration or epoch, and to monitor the overfitting phenomenon.

**Figure 14:**
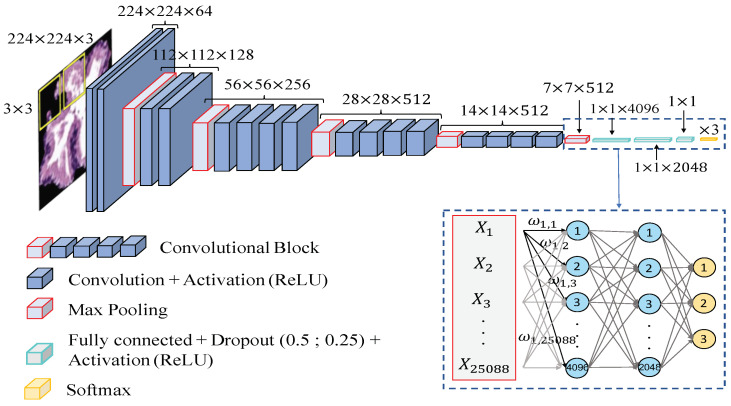
Network architecture used to construct predictive models from gland candidates of histopathological prostate images.

**Network architecture**. In order to compare the results achieved from the hand-driven learning approach, we make use of a very popular neural network called *Very Deep Convolutional Networks for Large-Scale Image Recognition (VGG19)*, which was proposed by Krizhevsky et al. [40] to face the challenge ILSVRC-2012 [41]. It is important to remark that Simonyan and Zisserman [42] modified some hyperparameters of this architecture with the aim of reducing the classification error from the images of the ImageNet [43] data set. They discovered that the performance of the neural network improved by moving each filter of the convolutional layer along the image by means of a sliding window with a small step size (stride). In addition, the authors achieved better results using smaller receptive fields, i.e., smaller sets of pixels inside the sliding window in each epoch. For these reasons, they designed the convolutional layers using receptive fields of size 3×3 and a stride s=1, instead of 11×11 and s=4 as [40]. The researchers developed six different CNN architectures with the same basis and modified the depth in each of them. Finally, they proposed the VGG16 and VGG19 to face the ImageNet 2014 challenge and reached the first and the second places in the localisation and classification tracks. In this work, we build the base model of the VGG19 neural network also using 3×3 receptive fields and a stride s=1, as shown in Figure 14. However, we include some modifications in the classification stage (a.k.a top model) with respect to the original architecture. Specifically, we also define two fully-connected layers, but we apply dropout layers, with a coefficient of 0.5 and 0.25 after each fully-connected layer, respectively. Note that these dropout layers allow us to avoid overfitting by means of randomly disconnecting the 50% and the 25% of the neurons, respectively. In addition, we define the second hidden layer with 2048 neurons, instead of 4096 as the original network, and we modify the output layer establishing only three neurons to address the multi-class problem between artefacts, benign glands and pathological glands (see Figure 14).

Additionally, due to the number of gland candidate instances of our database, it is insufficient to train from scratch an architecture with a such high depth, and we make use of the fine-tuning technique [44] to transfer the specific knowledge of the problem under study. This procedure allows for taking into advantage the previous wide knowledge acquired by the neural network VGG19 when it was trained on the *ImageNet* data set, which is composed of around 14 million of natural images belonging to 1000 different classes. In this way, we load the pre-trained weights ω and apply a *deep fine-tuning* [45] strategy. It should be noted that we only freeze the coefficients of the three first convolutional blocks using the pre-trained weights, whereas we retrain the coefficients of the last convolutional block with the specific images corresponding to the gland candidates. During the training phase, the update of the filter weights is performed in a similar way as before in the MLP case, but instead of updating them after each sample, now we set a batch size b=16 to recalculate them when 16 images are forward-propagated. At that moment, an error based on the categorical cross-entropy loss function Equation (37) is calculated to perform the back-propagation step updating the weights according to the Stochastic Gradient Descent (SGD) mechanism Equation (38). Note that here we also use a momentum γ=0.98 to improve the convergence rate of the CNN. A learning rate $\eta =10^{-5}$, a maximum number of epochs N=100 and a stop criterion of 15 epochs are also defined during the training process:

(38) ω(t+1)=ω(t)+V(t+1),

where

(39) $$V\left(t+1\right)=\gamma V\left(t\right)-\eta \frac{d}{d\omega }L\left(y,\hat{y}\right).$$

Finally, it is important to highlight the use of the *data augmentation* technique [46], with the aim of increasing our database of specific images. This technique allows for creating artificial samples similar to the originals by means of performing geometric and intensity transformations. In particular, we define the aggressive factor ratio as t=0.02 to create the synthetic samples with the same label class as the samples from which they were generated.

## 4. Results

**Figure 15:**
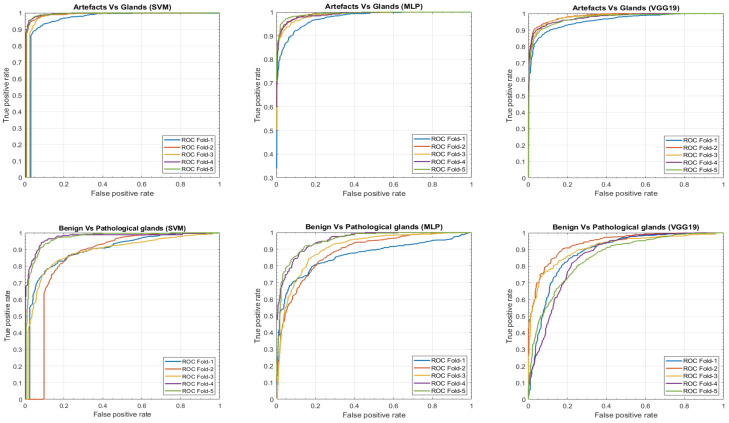
ROC curves achieved from the different hand-driven and deep-learning classifiers after evaluating the artefacts vs. glands problem and benign vs. pathological glands’ classification.

In this section, we present a comparison of the results achieved, on the one hand, by means of the hand-driven learning approach after performing a novel hand-crafted feature extraction, and, on the other hand, by means of a deep-learning strategy directly applied for the first time on gland candidate images. In particular, we evaluate the 5-trained models over each 5-cross-validation data sets. Table 2 reports the average results using following different figures of merit: sensitivity, specificity, positive predictive value (PPV), negative predictive value (NPV), F-score, area under the ROC curve (AUC) and accuracy. In addition, we also report the ROC curves (see Figure 15) with the aim of showing the discriminatory ability of the proposed classifiers according to each problem. Note that, despite the building of predictive models based on a multi-class classification approach, we expose the results distinguishing between artefacts and glands, and between benign and Gleason grade 3 glands.

It is also remarkable that this work is one of the few that provides results per gland candidate, instead of per patch. For this reason, we expose in Table 3 the results achieved by our best predictive model to compare them with those reached by other authors who also address this type of classification per gland unit. It should be remarkable that we perform an indirect comparison due to the private character of the code and databases used in [16,17].

In order to elucidate the differences between the performance of the three classification methods, we carry out a statistical analysis similar to the developed in SubSection 3.3.2. In this case, we calculate for each test data set not only the prediction labels, but also the scores, i.e., the probabilities of each gland candidate of belonging to a specific class. The aim is to determine the discriminatory ability of the different classification models taking into account the scores and the ground-truth labels. Particularly, for each classification model (SVM, MLP and VGG19), we address the achieved scores like a variable in order to study its independence level with respect to the targets. First, we perform a *Kolmogorov–Smirnov test* to determine if the variable under study follows a normal distribution of mean μ=0 and standard deviation σ=0 or not. Depending on it, we make use of the *ANOVA* or *Kruskal–Wallis* test, comparing the mean or median of such variable with respect to the categorical classes. These tests report a *p*-value for each possible class that allows for measuring the performance of each classifier when predicting the label of such class. We repeat this process five times (one per partition) and report the averaged *p*-values to show the differences between the classifiers (see Table 4).

**Figure 16:**
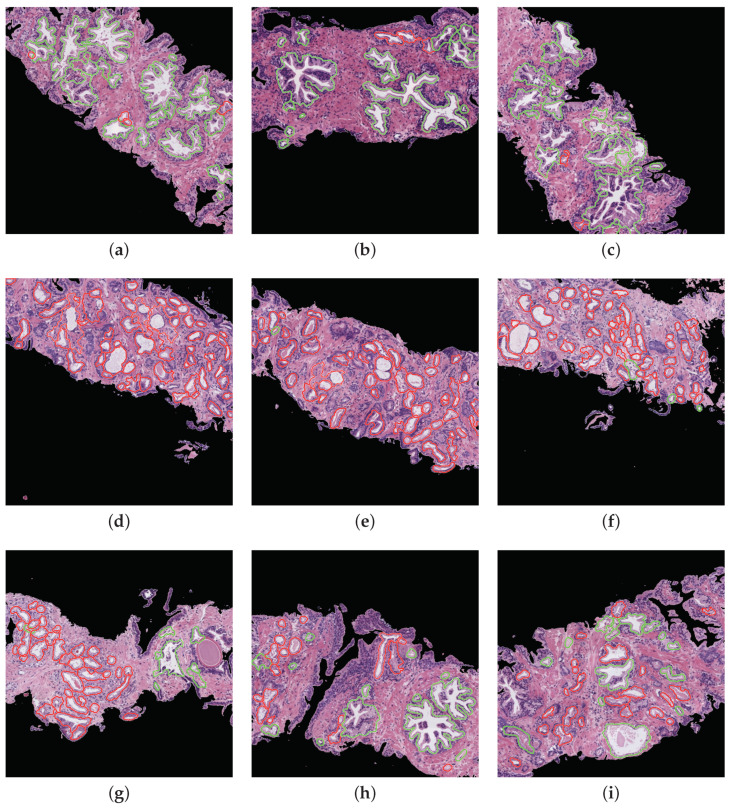
Automatic prediction of labels for each gland candidate, showing in green and red the labels corresponding to benign and pathological glands, respectively. (**a–c**) samples of 1024x1024 characterised by presenting a fully benign pattern, according to the diagnosis of an expert on pathological anatomy. (**d–f**) samples corresponding to a fully pathological pattern. (**g–i**) samples diagnosed with a combined benign and pathological pattern.

**Prediction phase**. With the aim of performing an external evaluation of our predictive model, we make use of histological samples of new patients from which we only know if they are healthy or suffer for prostate cancer in an initial stage, but we do not have a ground truth per gland. Particularly, we select the best classification models to perform a committee of evaluation in charge of predicting the label of the new samples. The final objective is to help the pathologists by means of an automatic system able to identify benign and pathological glands from histological prostate images. In Figure 16, we expose the prediction carried out for some 1024×1024 representative samples from different patients, in which we remark the automatically segmented glands according to the prediction carried out by the trained models. In particular, the boundary of the glands predicted as healthy are highlighted in green, whereas the contours of glands predicted as pathological are marked in red. It is important to remark that an expert pathologist diagnosed the samples reported in Figure 16 as: fully benign pattern (Figure 16a–c), fully pathological pattern (Figure 16d–f) and combined pattern (Figure 16g–i).

**Computational cost**. In Table 5, we list the average and standard deviation corresponding to the temporal intervals spent in each of the four processes that compose the end-to-end algorithm (clustering, segmentation, feature extraction and prediction) when analysing each patch.

Table 5 shows that, after studying the 854 healthy patches, the time of processing one of them is 38.68±34.44 seconds, in average terms. On the contrary, the average computational cost to automatically examine one of the 614 pathological patches is 18.87±26.12 seconds. Note that the time analysis was performed on an Intel i7 @4.00 GHz of 16 GB of RAM (Santa Clara, CA, USA) with a Titan V GPU (NVIDIA Corporation, Santa Clara, CA, USA). The classification strategies related to the SVM and MLP models were executed in MATLAB 2018b (MathWorks Corporation, Natick, MA, USA), whereas the methodology based on the VGG19 architecture was implemented in Python 3.5, using Keras framework with Tensorflow as backend. It should be remarkable that the final goal of the developed computer-aid system is to identify regions susceptible to be cancerous, with the aim of improving the effectiveness of experts by reducing the time invested in performing the visual examination.

## 5. Discussion

From Table 2 and Table 4, we observe that the hand-driven learning approach, using a Support Vector Machine (SVM) classifier with a nonlinear kernel of second order, seems to provide a very slight superiority of the results both for distinguishing between artefacts and glands and between benign glands and Gleason grade 3 glands. However, the differences between the three approaches are not evident enough to claim the outperforming of a specific classification model over the rest. Actually, according to Table 2, the results achieved from the VGG19 architecture decline a bit compared to the SVM and MLP classifiers, which may be due to the exhaustive and robust hand-crafted feature extraction and selection carried out during the hand-driven learning approach.

Notably, the values obtained for all figures of merit in Table 2 are similar in the first binary classification (i.e., artefacts vs. glands), but the differences are more prominent when discriminating between normal and cancerous samples. This is an understandable fact taking into account that artefacts and glands present very different contextual circumstances because artefacts are not usually surrounded by cytoplasm and nuclei components, unlike the lumen of the glands. Nevertheless, artefacts could be confused with lumen structures when an accumulation of glands envelop specific broken areas of tissue, which have the same colour as the lumen objects. These broken areas result in artefacts (false glands) because the model associates the nuclei of the surrounding glands as the nuclei of such broken areas. In this case, the features extracted from artefacts and glands are more similar to each other, and it can entail an error in the prediction. It should be noted that artefact elements usually are involved by pixels corresponding to stroma components, as shown in Figure 9, which is decisive for the correct classification of the false glands, i.e., artefacts. The visible differences between artefacts and glands allow for successfully addressing the classification problem from both machine-learning and deep-learning approaches. For this reason, we can observe in Figure 15 (bottom row), beyond the numerical results exposed in Table 2, that the performance of the three learning strategies is closely similar when the ROC curves are computed. Otherwise, the distinction between benign and pathological glands is more complex since both correspond to moderately differentiated carcinomas. In fact, we can appreciate in Figure 15 how the performance of the classifiers falls due to the similarities of the benign and pathological glands.

It is convenient to remember at this point that the predictive models are trained and evaluated, in each of the five iterations, making use of different samples attending to the medical history of the patients. This fact can introduce a slight diversity related to the structure and morphology composition of the histological images of the patients, so the results achieved when evaluating each data subset (fold) can differ from the rest. This phenomenon is observed in Figure 15 during the discrimination between artefacts and glands, where the fold corresponding to the blue curve presents a slight decrease in the ROC curve. On the contrary, when the distinction between benign and pathological glands is addressed by means of the SVM classifier, an outperforming is reached for two specific folds corresponding to the purple and green curves, which allows for situating the SVM classifier as the best model to predict the gland labels of samples from new patients.

Note that it can be striking that the SVM classifier based on traditional machine-learning techniques reports better results than the proposed deep-learning methodology. However, this fact makes sense taking into account that CNNs have specific limits and drawbacks related to the orientation and spatial relationships between objects. In particular, the deeper convolutional layers of the CNN allow for extracting information corresponding to the curves of the elements, whereas the high layers are able to understand more complex features. However, CNNs do not take into account important spatial hierarchies between simple and complex objects. For this reason, the deep-learning approach successfully discriminates between artefacts and glands, since it finds differences in the composition of the images. Nevertheless, its performance considerably decreases when facing the benign vs. pathological problem, since the CNN is able to identify the shape of the glands and lumens, as well as their characteristic components (cytoplasm and stroma pixels), but it can not manage the differences concerning the spatial dimensions, orientations and sizes of the glands. In the problem under study, the size of the lumens and glands is really decisive to determine the class of a specific gland, as we explain in Section 1. Thus, due to the hand-driven learning approach allowing for taking into account the dimensions of the gland candidates in some of the hand-crafted features, it is coherent that the SVM classifier provides better results than the convolutional neural network.

Regarding the results exposed in Table 3, it should be remarkable that the comparison is performed in an indirect way since there is no public database of gland units to contrast with our results. Nevertheless, the approach presented in this work outperforms the methods proposed by other state-of-the-art studies that also follow an approach based on gland unit classification and evaluation. In particular, relating to the artefacts vs. glands problem, Xia et al. [17] did not report results because they did not implement an approach based on an initial lumen identification, but they classify all segmented glands into two classes: benign and pathological. On the contrary, Nguyen et al. [16] also considered the artefact elements and reported in its work an accuracy of 0.93 in the discrimination between artefacts and glands. Note that we improve their results exceeding its accuracy value by 1.6%. However, the real challenge lies in discerning between healthy glands and Gleason grade 3 glands. In this case, we provide the most relevant differences concerning the rest of the works. Specifically, we report increases of 2.3% and 9.3% with respect to the studies proposed by Xia et al. [17] and Nguyen et al. [16], respectively. Also, we surpass the multi-class accuracy reached in [16] with 10.6%.

Regarding the simulation of the clinical practice, we show in Figure 16 the great ability of the proposed computer-aid system to identify and discriminate the healthy and pathological gland units, which are highlighted in green and red, respectively. We can determine that the final proposal of our work is successfully performed attending to the example reported in Figure 16, which demonstrates that we can provide to pathologists an automatic model capable of accurately detecting specific areas susceptible to be cancerous. Making use of this visual prediction system, we can help the experts in the diagnosis task reducing its workload.

Finally, concerning the computational cost, it is worth noting that the automatic analysis of pathological patches requires half the time compared to the healthy images. This fact is mainly propitiated by the segmentation stage, which takes more time to analyse benign glands due to its larger size, as well as its fusiform appearance. Paying attention to the temporal intervals (Table 5), we can observe high standard deviation values mainly reported in the segmentation and feature extraction stages. This fact occurs since the computational cost is closely related to the number of glands in each patch, i.e., the more number of glands per image, the more time is necessary to segment and to extract features from them.

## 6. Conclusions

This paper proposes two novel approaches to automatically identify the first stage of prostate cancer from images of gland candidates previously segmented. In the first approach, we perform, in a novel way, a combination of four kinds of descriptors based on morphology, texture, fractals and contextual information for addressing the hand-crafted feature extraction stage. In addition, advanced machine-learning classifiers are optimised to face the problem from a nonlinear point of view. Regarding the second approach, we present a convolutional neural network built upon VGG19 to automatically extract the relevant features from artefacts, benign glands and pathological glands to subsequently address the multi-class discrimination problem. The hand-driven learning approach making use of the SVM with a quadratic kernel provides the best classification results and also outperforms the most relevant methods proposed in the state of the art. It should be noted that we obtain satisfactory results when testing the trained models with samples of new patients. However, it would be necessary to carry out additional tests, as well as retrain the predictive models making use of a larger data set, to incorporate this system in a clinical environment. It is worth noting that an increase in the database could improve the performance of the classification models, mainly those based on deep learning algorithms. In future research lines, we propose not only to improve the results of the VGG19 architecture through making use of other popular state-of-the-art CNNs (e.g., ResNet, DenseNet, Xception, etc.), but also to design novel CNN architectures to be trained from scratch using an enlarged gland database. Additionally, we also propose, as one of the main future directions, the design of a computer-aid system capable of addressing the identification of any pathological grade of the Gleason score scale.

## Figures and Tables

**Table 1:** Features selected after applying the statistical analysis.

**Morphological features (13)**	*Gland (6)*	$G_{area}$, $G_{eccent}$, $G_{equivDiam}$, $G_{extent}$, $G_{solidity}$, $G_{roundness}$
	*Lumen (7)*	$L_{area}$, $L_{eccent}$, $L_{equivDiam}$, $L_{extent}$, $L_{perimeter}$, $G_{solidity}$, $G_{roundness}$
**Fractal analysis (11)**	*Cyan (3)*	$H_{cyan}^{30{}^{\circ}}$, $H_{cyan}^{45{}^{\circ}}$, $H_{cyan}^{60{}^{\circ}}$
	*Hematoxylin (5)*	$H_{hmtx}^{0{}^{\circ}}$, $H_{hmtx}^{30{}^{\circ}}$, $H_{hmtx}^{45{}^{\circ}}$, $H_{hmtx}^{60{}^{\circ}}$, $H_{hmtx}^{90{}^{\circ}}$
	*Eosin (3)*	$H_{eosn}^{30{}^{\circ}}$, $H_{eosn}^{45{}^{\circ}}$, $H_{eosn}^{60{}^{\circ}}$
**Textural features (94)**	*Cyan (32)*	$Homo_{nGLCM_{C}^{0}}$, $Cont_{nGLCM_{C}^{0}}$, $Ener_{nGLCM_{C}^{0}}$, $Corr_{nGLCM_{C}^{0}}$, $\mu_{nGLCM_{C}^{0}}^{j}\left(1-3;7-8\right)$, $Homo_{nGLCM_{C}^{45}}$, $Cont_{nGLCM_{C}^{45}}$, $Corr_{nGLCM_{C}^{45}}$, $LBP_{8,1}^{riu2}\left(1-10\right)$, $LBPV_{8,1}\left(1-10\right)$
	*Hematoxylin (27)*	$Homo_{nGLCM_{H}^{0}}$, $Ener_{nGLCM_{H}^{0}}$, $Corr_{nGLCM_{H}^{0}}$, $\mu_{nGLCM_{H}^{0}}^{j}\left(1-2;7\right)$, $Corr_{nGLCM_{H}^{45}}$, $LBP_{8,1}^{riu2}\left(1-10\right)$, $LBPV_{8,1}\left(1-10\right)$
	*Eosin (35)*	$Homo_{nGLCM_{E}^{0}}$, $Cont_{nGLCM_{E}^{0}}$, $Corr_{nGLCM_{E}^{0}}$, $\mu_{nGLCM_{E}^{0}}^{j}\left(1-8\right)$, $Homo_{nGLCM_{E}^{45}}$, $Cont_{nGLCM_{E}^{45}}$, $Corr_{nGLCM_{E}^{45}}$, $Ener_{nGLCM_{E}^{45}}$, $LBP_{8,1}^{riu2}\left(1-10\right)$, $LBPV_{8,1}\left(1-10\right)$
**Contextual features (18)**	*Nuclei (9)*	$nuclei_{BB}^{num}$, $nucleiRatio_{BB}^{num}$, $nuclei_{Gland}^{num}$, $nucleiRatio_{Gland}^{num}$, $nucleiRatio_{Gland-BB}^{num}$, $nuclei_{BB}^{pix}$, $nucleiRatio_{BB}^{pix}$, $nucleiRatio_{Gland}^{pix}$, $nucleiRatio_{Gland-BB}^{pix}$
	*Cytoplasm (5)*	$cyto_{BB}^{pix}$, $cytoRatio_{BB}^{pix}$, $cyto_{Gland}^{pix}$, $cytoRatio_{Gland}^{pix}$, $cytoRatio_{Gland-BB}^{pix}$
	*Lumen-Nuclei-Cytoplasm (4)*	$Ratio_{L-G}^{pix}$, $\mu_{L-Edge}$, $\sigma_{L-Edge}$, $RatioTor_{C-N}$

**Table 2:** Classification results per gland candidate. (**PPV**–Positive Predictive Value; **NPV**–Negative Predictive Value; **AUC**–Area Under the Receiver Operating Characteristic (ROC) Curve).

	*Artefacts vs. Glands*			*Benign vs. Pathological*		
	SVM	MLP	VGG19	SVM	MLP	VGG19
**Sensitivity**	0.945±0.016	0.930±0.011	0.901±0.020	0.802±0.058	0.802±0.079	0.747±0.109
**Specificity**	0.952±0.026	0.952±0.028	0.939±0.026	0.873±0.073	0.819±0.094	0.780±0.123
**PPV**	0.911±0.058	0.913±0.053	0.884±0.059	0.845±0.069	0.796±0.084	0.779±0.101
**NPV**	0.964±0.018	0.956±0.019	0.945±0.016	0.843±0.052	0.837±0.059	0.769±0.047
**F-Score**	0.927±0.029	0.921±0.030	0.891±0.032	0.820±0.030	0.793±0.014	0.753±0.059
**AUC**	0.984±0.011	0.987±0.007	0.974±0.010	0.922±0.045	0.912±0.042	0.889±0.036
**Accuracy**	0.946±0.017	0.943±0.030	0.925±0.018	0.883±0.026	0.853±0.020	0.817±0.031

**Table 3:** Accuracies comparison with other state-of-the-art studies performed at the gland level.

	Xia et al. [17]	Nguyen et al. [16]	Proposed Model
**Artefacts vs. Glands**	-	0.93±0.04	0.946±0.017
**Benign vs. Pathological**	0.86±0.02	0.79±0.08	0.883±0.026
**Multi-class classification**	-	0.77±0.07	0.876±0.026

**Table 4:** Average of *p*-values achieved after calculating the independence level between the probability of each class and the targets, from each classifier.

	Artefact	Benign Gland	Pathological Gland
**SVM**	$4.2813\times 10^{-223}$	$3.3720\times 10^{-118}$	$2.1903\times 10^{-210}$
**MLP**	$6.3328\times 10^{-203}$	$2.8052\times 10^{-143}$	$7.3978\times 10^{-195}$
**VGG19**	$3.9919\times 10^{-220}$	$1.2796\times 10^{-137}$	$5.8481\times 10^{-192}$

**Table 5:** Time average and standard deviation (in seconds) that each process requires to determine the benign or pathological pattern of patches of 1024×1024.

	Healthy Tissues (s)	Cancerous Tissues (s)
Clustering stage	2.79±0.58	2.39±0.39
Gland segmentation	25.24±29.63	9.69±18.99
Feature extraction	9.75±7.75	5.88±7.48
Prediction	0.04±0.01	0.04±0.01
**End-to-end algorithm**	38.68±34.44	18.87±26.12
